# Supplementary material for: Effect of government’s environmental attention on corporate philanthropy based on the institutional theory: Evidence from China’s heavily polluting companies
Source: PLoS One. 2024 Oct 22;19(10):e0309595. doi: 10.1371/journal.pone.0309595 (PMC11495631; doi:10.1371/journal.pone.0309595)
Supplement: Attachment — Submitted filename: Response to Reviewers.docx [file pone.0309595.s002.docx]

Dear Reviewers,

I hope this letter finds you well. I am writing to express my gratitude for the thorough reviews of my manuscript. I am grateful for the time and effort reviewers have dedicated to providing valuable feedback that has significantly enhanced the quality of my research.

I have carefully reviewed the comments and suggestions from two reviewers, and have made the necessary revisions to address each point. I believe that these changes have strengthened the paper's theoretical framework, methodological approach, and empirical findings. Below is a summary of the revisions I have made.

|  | **Reviewer-1** | |
| --- | --- | --- |
|  | **Observations** | **Reply** |
| 1 | The innovation of this paper needs to be highlighted in the abstract. | We have emphasized the innovation of this paper in the abstract. Please see Para 1 at Page No.2 |
| 2 | The author should summarize the existing research gaps and highlight the innovation of this paper after completing the literature review. | From the perspective of political motivations of corporate philanthropy in the Chinese context, we have revisited the literature review and extensively rewritten the introduction. We have analyzed the shortcomings of relevant literature, summarized the gaps in existing research, and highlighted the innovation of this paper after presenting the main conclusions. Please see Para2 at Page NO.9 and Page 12-14. |
| 3 | In the second section, please provide a framework diagram of this paper. | We have supplemented a framework diagram of this paper. Please see Page 22 |
| 4 | What are the managerial insights/policy implications of this study? Compared with available literature, what are the theoretical contributions and application values of this study? It is suggested to enhance the corresponding discussions in the conclusion part. | We have expanded the discussion of the theoretical contributions and policy implications of this study in the conclusion part. Please see Page 51-53. |
| 5 | Whether in terms of structure or content, this article should reflect more scientific problems, research status, research methods and innovation | This paper has undergone almost comprehensive revisions to the introduction, theoretical analysis, method, discussion of mechanism, and the conclusion, with adjustments made to the structure. In particular, the introduction has been rewritten following a logical flow of background, research problems, current research status, research methods, main conclusions, and innovations. The other sections have also undergone significant modifications. Now this paper could reflect more scientific problems, research status, research methods, and innovations. |
| 6 | The article lacks an important discussion link, in which the author should focus on describing the differences between the article study and other scholars' studies, thus highlighting the relevance and academic value of the article, the following literature should be helpful for your research: (1) Reduction pathways identification of Agricultural Water Pollution in Hubei Province, China. (2) A differential game of water pollution management in the trans-jurisdictional river basin. (3) Coordination of the Industrial-Ecological Economy in the Yangtze River Economic Belt, China. | Following the style of several recommended literature, we have added a comparative analysis of differences between this study and other scholars' studies, focusing on the unique theoretical contributions of this paper. Please see Page 12-14. |
| 7 | Please do a thorough check on the English quality of this paper | We have thoroughly reviewed and improved the English quality of this paper. |
| 8 | The structure of the article needs to be greatly adjusted, and it is recommended that the author read more relevant papers in journals | After reading many relevant papers in journals, we have made substantial adjustments to the structure of this paper. |
| 9 | The article was not written following the correct journal's guidelines to be considered for publication (INTORDCUTION→MRTHOD→RESULTS→DISSCUSION→CONCLUSION). | Following the correct journal’s guidelines, we have adjusted the structure of this paper in the order of "Introduction (and Theory Analysis) → Method→ Results→ Discussion→ Conclusion". |
|  | **Reviewer-2** | |
| 1 | The effort made in the literature for this manuscript is insufficient. This requires a systematic analysis of the motives behind corporate donation behavior and the influence of government factors. Furthermore, there is no updated literature for this manuscript. | From the perspective of non-transactional political motivations of corporate philanthropy and the impact of campaign-style institutional uncertainty, we have updated numerous latest references, reorganized the literature review, and conducted a systematic theoretical analysis. Please see the rewritten introduction at Page 3-14. |
| 2 | Why is the research focused on corporate donations rather than corporate environmental investment. This manuscript needs to present its theoretical basis and contributions from this perspective. | In the Chinese context, the government plays a dominant role in shaping corporate behaviors and granting legitimacy. Corporate philanthropy, a non-productive corporate behavior, is the most important strategic tool for Chinese companies to manipulate legitimacy and respond to institutional uncertainty. Therefore, this paper focuses on corporate philanthropy rather than corporate environmental investment, which is with productive purposes. From this perspective of institutional theory, this paper proposes a framework of theoretical analysis. Please see Para 1-2 at Page No.6 and Page 12-14. |
| 3 | Theoretical analysis lacks support. This is mainly the author's viewpoint, rather than an extension of existing classical theories. In addition, this suggestion provides a framework diagram of the relationship between classical theories, theoretical extensions, and research hypotheses. | We have rewritten the theoretical analysis part, analyzing the institutional environment and the problems faced by Chinese companies, extending the classical theory of motivations of corporate philanthropy. We have also added a framework diagram to facilitate understanding of the relationship between the theoretical basis, theoretical extensions, and research hypotheses in this paper. Please see Page 17-22. |
| 4 | Formula (1) does not introduce the relevant variables. In addition, there is a complete multiple contribution between dummy variables and fixed effects, and some dummy variables are not needed. | We have added the introduction to relevant variables in Model (1). Since some unnecessary dummy variables will be absorbed by industry and year fixed effects, we have removed these dummy variables from Model (1). Please see Para 3 at Page 24, Para 1 at Page 25, and Page 27-29. |
| 5 | Some variables in the control variables are nominal variables. This may present challenges. | Although firm size and logarithm of monetary cash are nominal variables in the control variables, there are only two nominal variables in the multiple regression model, which may cause minor interference. Furthermore, Model (1) controls for year fixed effects, which we believe could alleviate potential issues to some extent. |
| 6 | The mechanism lacks persuasiveness. | We have re-discussed the mechanism and proposed that the mechanism should be expectation changing, which has been validated from the perspectives of the intensity of changes and the sensitivity of companies to the change. We believe this mechanism is reasonable, consistent with the revised theoretical framework, and has persuasiveness. Please see Page 43-45. |

I have also made several other minor adjustments to improve the clarity and readability of the study. I have included the revised version of the paper, labeled as "Revised Manuscript with Track Changes " for your review.

Thank you once again for your valuable comments and suggestions. I hope that the revised paper will meet the standards and expectations of the journal and be considered for publication.

Best regards,

Dongli Cao
